# Acknowledgment to the Reviewers of *Microorganisms* in 2022

**DOI:** 10.3390/microorganisms11010227

**Published:** 2023-01-16

**Authors:** Microorganisms Editorial Office

**Affiliations:** MDPI AG, St. Alban-Anlage 66, 4052 Basel, Switzerland

High-quality academic publishing is built on rigorous peer review. *Microorganisms* was able to uphold its high standards for published papers due to the outstanding efforts of our reviewers. Thanks to the efforts of our reviewers in 2022, the median time to first decision was 15 days and the median time to publication was 35 days. Regardless of whether the articles they examined were ultimately published, the editors would like to express their appreciation and thank the following reviewers for the time and dedication that they have shown *Microorganisms*:
Abashina, Tatiana N.Light, SamAbdalhamed, Abeer MostafaLikar, MatevzAbdalla, HenriqueLikhoshway, Yelena ValentinovnaAbdel Moneim, Ahmed E.Lilia, MateiAbdelaal, KhaledLim, SangyongAbdel-Glil, MostafaLin, Chang-ChiAbdelhamed, HossamLin, HaishuAbdelmegeid, Mohamed K.Lin, Hong-Ting VictorAbe, HarukaLin, Hsing-JuhAbia, Akebe Luther KingLin, Wan-RouAboelhadid, Shawky M.Lin, WeichihAboellail, Tawfik A.Lin, Xin-TangAbo-elyousr, KamalLinares-Pastén, Javier A.Abo-Elyousr, Kamal A. M.Lindbäck, TorilAbolhasani, MiladLinden, SaraAbomoelak, BassamLine, John EricAbouloifa, HoussamLing, JiqiangAbraham, RachyLinial, MichalAbraham, Wolf RainerLinke, DirkAbras, AlbaLiovic, IvicaAbrouk, DanisLipatova, IndrėAbulila, AmroLiptáková, AdriánaAcedo, JeellaLis, MagdalenaAchinelly, Maria FernandaLisiecka, UrszulaAcosta Gutiérrez, RoxanaLiu, BinAdamakis, Ioannis-DimosthenisLiu, DongAdamcová, DanaLiu, GuangxiuAdamczuk, MałgorzataLiu, GuoxiangAdamczyk-Popławska, MonikaLiu, HeAdams, Leslie GarryLiu, HongchangAdams, MerrinLiu, Po-YuAdamus-Białek, WiolettaLiu, QingAdégbitè, Bayodé RoméoLiu, Wei-chungAdhikari, ArjunLiu, XiaojunAdnan, MohdLiu, XiaoluAdnan, MuhammadLiu, Xiao-YongAdriana, PauceanLiu, Yung-ChuanAfonin, AlexeyLivingston, ElizabethAggarwal, AnupriyaLjaljevic Grbic, MilicaAggarwal, SomyaLlama-Palacios, AranchaAgudelo-Romero, PatriciaLlamas, AngelAgudelo-Suárez, Andrés AlonsoLleo, MarAguilar‑Pontes, Maria VictoriaLo Giudice, AngelinaAguilar-Zarate, PedroLo Monaco, AngelaAguilera, MargaritaLo, Shih-YenAguirre-Medina, Juan FranciscoŁobacz, AdrianaAhmad, LukmanLobus, NikolayAhmadi, MirelaLoewy, ZviAhmed, ShabbirLoeza-Lara, PedroAhmed, ShamimLogue, CatherineAhn, JuheeL’Ollivier, CoralieAillot, LudovicLomakina, NataliaAizpurua, OstaizkaLombardelli, ClaudioAkinosoglou, KarolinaLong, JieAktepe, Turgut E.Longo, Maria A.Al-Ahmad, AliLopes, Bruno S.Alashaal, SaraLopes, MarleneAl-Asmakh, Maha A.Lopez, Blanca R.Albes, Johannes M.López, Lucía MéndezAlcázar-Fuoli, LauraLópez, MaríaAlcover, Maria MagdalenaLópez-Castro, JoséAl-Deeb, Mohammad AliLopez-Garrido, JavierAldred, KatieLópez-González, Juan A.Aldred, NicholasLópez-Medrano, RamiroAlduina, RosaLops, FrancescoAleksova, AnetaLorena, DediuAlessiani, AlessandraLorenzo-Morales, JacobAlexander, DavidLoster, Jolanta E.Alexander, GalushkoLosurdo, GiuseppeAlexandrova, AlinaLoureiro, RicardoAlexopoulos, AthanassiosLovecká, PetraAleynova, Olga A.Lower, BrianAlfonso, Jorge JavierLozada Nur, FrancinaAli, AkhtarLozo, JelenaAli, AshaqLu, Hai-LongAli, IslamLu, Mei-ChinAli, Syed AzmalLu, Ming-WeiAlisaac, EliasLu, Shi-EnAlizadeh, MohammadaliLubisi, Baratang A.Alizargar, JavadLucas, SebastianAlKadmy, IsraaLuchian, IonutAllen, AdrianLuciani, FilippoAllen, Edith B.Luciani, MichelangeloAllen, HerbertLuciani, MirellaAllmang, ChristineLucke-Wold, BrandonAl-Marzooqi, WaleedLuganini, AnnaAlmazán, ConsueloLuis, AnaAlmeida, RodrigoLukaszewski, RomanAl-Saman, Mahmoud A.Lukic, IvaAlshaikh, Belal N.Luna, JulianaAlsherif, Emad A.Lunestad, Bjørn T.Alsolaiss, JafferLuo, PengAltaf, Muhammad AhsanLuo, WentaiAltayeb, HishamLuo, YunlongAlvarez, Angel H.Luongo, VincenzoÁlvarez, DianaLuporini, PierangeloÁlvarez, María S.Luquez, CarolinaAlvarez-Martinez, Francisco JavierLurie-Weinberger, Mor NadiaÁlvarez-Mercado, Ana I.Lušić Vukić, DarijaAlvarez-Narvaez, SonsirayLuther King, Abia AkebeAmalfitano, StefanoLutz, PhilippAmaral, PriscillaLuvisi, AndreaAmaro, FátimaLyamlouli, KarimAmato, FeliceLyman, MeghanAmils, RicardoLysnyansky, InnaAmmar, El DesoukyMa, AnzhouAmmendolia, Maria GraziaMa, DongzhuAmoutzias, GrigorisMa, KesenAn, RanMacaluso, GiusiAna María, SandinoMachado, AntónioAnaissi, FauzeMachado, Daniela Marlene Da SilvaAnantpadma, ManuMaciver, SutherlandAnderson, Anne J.Maciver, Sutherland K.Anderson, O. RogerMacLeod, David A.Andjelkovic, SnezanaMacori, GuerrinoAndrade, GaldinoMad’ar, MariánAndré, MarcosMadigan, MichaelAndreani, Nadia AndreaMadigan, Michael T.Andreoni, FrancescaMadoroba, EvelynAndronov, Evgeny E.Maeda, IsamuAndrukhov, OlehMaekawa, ShunAndryukov, Boris G.Maestri, ElenaAngelucci, Clotilde B.Magariños Ferro, BeatrizAngiolella, LetiziaMagdalena, WoźniczkaAngulo, DavidMagiorkinis, EmmanouilAnita, AdrianaMagnano Di San Lio, GaetanoAnne, JozefMahan, MichaelAnnenkova, Nataliia V.Mahar, JackieAnniballi, FabrizioMahdi, MohamedAns, MuhammadMahdinia, EhsanAntonakopoulos, NikolaosMahnic, AleksanderAntonets, KirillMahony, Timothy JohnAntón-Herrero, RafaelMaicas, SergiAntoran, AitziberMaiese, AnielloAntunes, JorgeMaiolino, PaolaAppel, JensMajewska, AnnaArabatzis, MichaelMajoros, LaszloAraújo Filho, Renisson NeponucenoMajtan, JurajAraújo Junior, Joao P.Mak, Gannon C. K.Archbald-Pannone, LaurieMakádi, MariannaArciszewski, MarcinMakepeace, BenjaminArfuso, FrancescaMaksimova, Yuliya Gennad’evnaArienzo, AlyexandraMakuch, EdytaArmalyte, JulijaMalachova, KaterinaArmengol, RamónMalagnino, VincenzoArnalich, FranciscoMałajowicz, JolantaArndts, KathrinMalcangi, GiuseppinaArora, GunjanMalfeito-Ferreira, ManuelArora, Gurpreet KaurMałgorzata, ZiarnoArranz-Solís, DavidMalicka, MonikaArruebo, PilarMalik, AdeelArsenopoulos, KonstantinosMalinen, Anssi M.Artigas, MariaMałolepsza-Jarmołowska, KatarzynaArtyszak, ArkadiuszMaloney, JennyArukha, Ananta PrasadMaltman, ChrisArunachalam, KannappanMan, AdrianAsakawa, MitsuhikoManandhar, Krishna DasAsaturova, AleksandraMancha-Agresti, PamelaAshames, AkramMancheño, JoséAshhab, Ashraf AlMancianti, FrancescaAshrafali, Mohamed IsmailMändar, ReetAsnicar, FrancescoMandilara, GeorgiaAsperges, ErikaManfredi, MarcoAtamanalp, MuhammedManga, MusaAtanasov, VasilMangiagalli, MarcoAthanasakopoulou, ZoiManning-Cela, RebecaAthanasiou, Labrini V.Manoj-Kumar, ArthikalaAtlung, ToveManolescu, Loredana Sabina CorneliaAttia, YoussefMantzoukas, SpiridonAttila, GlatzManu, MinodoraAtukunda, PrudenceMara, ArlindAtwood, WalterMarangoni, AntonellaAubourg, Santiago P.Maraolo, Alberto EnricoAubry, AlexandraMarbán-Castro, ElenaAuchtung, JenniferMarçais, BenoitAuclair, EricMarchand, Patrice A.Audia, Jonathon P.Marchesi, IsabellaAudigé, AnnetteMarciniak-Łukasiak, KatarzynaAugustyniak, AdrianMarcinnò, AndreaAung, Meiji SoeMarcon, EricAurilia, VincenzoMarek, AgnieszkaAuvinen, PetriMargarit, ImmaculadaAwad, MohamedMargarita, ValentinaAwosile, BabafelaMaría Ángeles, Núñez SánchezAyala, JuanMaria, PapaleAyalew, Lisanework E.Mariita, Richard M.Aydin, MalikMarimon, Jose MariaAyyash, MutamedMarín, ClaraAzab, WalidMarín, ReinaldoAzinheira, Helena G.Marini, Herbert RyanAzinheiro, SarahMarini, MonicaAzzam, Mahmoud MostafaMarinković, JelenaAzzimonti, BarbaraMarino, RosannaB Gowda, Siddabasave B.Marinov, Georgi K.Babaei-Ghazvini, AminMariscal, VicenteBaba-Moussa, Lamine SaïdMarkiewicz, Lidia HannaBabkin, Igor V.Markovska, RumyanaBabkov, Denis A.Marmeisse, RolandBaccigalupi, LoredanaMarotta, FrancescaBach, EveliseMarques, CátiaBach, HoracioMarruchella, GiuseppeBąchor, RemigiuszMarsili, EnricoBachvaroff, Tsvetan R.Martellos, StefanoBadger-Emeka, LorinaMartin Pelaez, SandraBadosa, EstherMartín, Juan FranciscoBagi, AndreaMartin, RonnyBagi, FerencMartínez, Beatriz RomeroBahar, Ali AdemMartinez, ElenaBahrim, GabrielaMartínez, José LuisBai, NanMartínez-Espinosa, Rosa MaríaBailey, Adam LeeMartínez-Hernández, FabiánBajagain, RishikeshMartino, Piera AnnaBajer, AnnaMartins, IvoneBakiu, RigersMartin-Torrijos, LauraBalaguera-López, Helber EnriqueMartirosian, GajaneBaldin, ClaraMartu, AlexandraBaldy-Chudzik, KatarzynaMartyniuk, StefanBalestra, GiorgioMarutescu, Luminita GabrielaBaliere, CharlotteMarzec, MichałBalmas, VirgilioMarzec-Grządziel, AnnaBalogh, PéterMasatani, TatsunoriBalotf, SadeghMashkova, Irina V.Baloyi, JosephMaslennikov, RomanBaltoumas, FotisMaslov, MikhailBamford, ConnorMassimino Cocuzza, Giuseppe ErosBamias, GiorgosMastore, MaristellaBamisile, Bamisope SteveMastrochirico Filho, Vito AntonioBanach, ArturMasuya, HayatoBañares, AngeloMatarese, GiovanniBanchi, ElisaMatei, FlorentinaBand, Victor I.Matei, IoanaBandín, IsabelMateos-Hernández, LourdesBandini, GiuliaMathai, PrinceBanerjee, DeepanwitaMathew, BasilBanerjee, GoutamMathew, RenyBanerjee, RajdeepMathews, Patrick D.Banerjee, SrijonMathison, Blaine A.Bánvölgyi, AndrásMatiašovic, JánBao, JunMatny, OadiBaptista, RodrigoMatsubara, RyumaBaraniak, AnnaMatsushita, KazunobuBaranowski, EricMatsuura, KatsumiBaráth, ZoltánMatthews, G. A.Barbalho, SandraMatthiessen, BirteBarbarossa, AlexiaMattila, SariBarbero, Rondineli PavezziMatulić, DanielBarbosa, Helene SantosMatúš, ČomaBarcyté, DoviléMatys, JacekBardosova, MariaMauk, Michael G.Bargiela, RafaelMaurya, Akhilendra KumarBar-Haim, ErezMavrogianni, Vasia S.Baril, PatrickMayo, BaltasarBarinova, SophiaMayr, AstridBarker, BridgetMazei, Yuri A.Barlaam, AlessandraMazur, AndrzejBarquera, BlancaMazur, MarcelinaBarr, Kelli L.Mazzacane, SanteBarros-Battesti, Darci MoraesMazzarello, VittorioBarrow, PaulMazzawi, TarekBartholomäus, AlexanderMcDonald, Mary RuthBartie, KerryMcGee, LesleyBarton Behravesh, CaseyMcGrath, JohnBarton, WileyMcGregor, Bethany L.Bartošová-Sojková, PavlaMcLean, Kyle J.Bartoszewicz, MarekMcLean, RobertBartrand, TimothyMcMillan, ElizabethBarua, NilakshiMcMorran, BrendanBasco, LeonardoMcMurray, MichaelBasen, MirkoMcPhee, Joseph B.Basmaci, RomainMead, Jan R.Batrinou, AnthimiaMedina-Carmona, EncarnacionBauer, CarlMedo, JurajBaum, ChristelMedveďová, AlžbetaBavaro, DavideMeftah Kadmiri, IssamBavikatte, GaneshMehdizadeh Gohari, ImanBayart, Jean-LouisMei, HanBayer, KarlMeir, MichalBayer, WibkeMelekhina, Elena N.Baymakova, MagdalenaMelhem, MárciaBeard, MatthewMelis, AnastasiosBeaver, Jordan W.Mello, Thaís P.Beck, ReljaMelo, Luís F.Becker, Donald F.Mendes, RafaelBecker, SörenMendonça-Lima, LeilaBedenić, BrankaMenéndez, EstherBehar, AdiMeneses, Lisandra RochaBehnam, Mira A. M.Menna-Barreto, Rubem Figueiredo SadokBehnke-Borowczyk, JolantaMensitieri, FrancescaBeitz, DonaldMerenkova, SvetlanaBej, AsimMerino, Patricio RetamalBelbahri, LassaâdMeroni, GabrieleBełcik, AnetaMešić, ArminBeld, JorisMesplède, ThibaultBeliavskaia, AlexandraMessaritakis, IppokratisBelkova, NataliaMessi, PatriziaBelluco, SimoneMessyasz, BeataBelov, Andrey A.Meštrović, TomislavBeltrame, AnnaMetcalf, JamesBeltran, JesusMeto, AidaBelyi, YuryMetwally, GalalBelykh, OlgaMeziti, AlexandraBencúrová, ElenaMiccoli, AndreaBender, KellyMichalski, MirosławBenić, MiroslavMiddelveen, MarianneBenítez Rodríguez, RocíoMiele, Adriana E.Benìtez, EmilioMierzejewska, JolantaBenítez-Mateos, Ana I.Miftode, Ionela-LarisaBennett, John E.Miglė, GabrielaitėBentivegna, EnricoMigliara, GiuseppeBercovier, HerveMignolet, JohannBerdalet, ElisaMiguel, DaniloBerestetskiy, Alexander O.Mihasan, MariusBergamo, GreiciMiklasińska-Majdanik, MariaBerger, MassimoMilanova, AneliyaBergh, ØivindMilanovic, ZoranaBergholz, Teresa M.Milanowski, Łukasz M.Berisio, RitaMilicevic, JelenaBerlot, GiorgioMiller, Aaron W.Bermudes, DavidMiller, AnaBernadette-Emoke, TelekyMiller, Catherine M.Bernard, NicoleMiller, Scott R.Bernardazzi, ClaudioMilner, DavidBernardi, SaraMiloshev, GeorgeBernotienė, RasaMin, HangBertelloni, FabrizioMin, ZawBerthold-Pluta, AnnaMinami, MasaakiBertolini, CamillaMinard, PhilippeBertzbach, Luca DaniloMinotti, ChiaraBęś, AgnieszkaMiran, WaheedBesaury, LudovicMiranda Marques, LucasBetekhtin, AlexanderMiranda, Jose M.Bethunaickan, RamalingamMiranda, LuisaBettoni, Jean CarlosMirończuk, Aleksandra M.Bevilacqua, AntonioMironeasa, SilviaBeyer, KatharinaMironov, VladimirBezirtzoglou, EugeniaMišak, Zrinjka MatekBezza, Fisseha AndualemMisal, Santosh A.Bharat, AmritaMishra, ArchanaBhargava, AditiMishra, BharatBhat, Sartaj AhmadMishra, Hridesh KumarBhatia, Shashi KantMishra, MeerambikaBhatt, PankajMitarai, SatoshiBhattacharjee, MrinalMitchell, GrahamBhattacharyya, TapanMiteva, Lyuba DinevaBhinderwala, FatemaMitraparp-Arthorn, PimonsriBiadała, AgataMitrea, LauraBiamonte, FilippoMitsou, EvgeniaBianco, ArmandodorianoMiura, HirokiBianco, FrancescoMiyasaka, HitoshiBiassoni, RobertoMiyashita, NaoyukiBidet, PhilippeMiyazaki, KoujiBielan, ZuzannaMizuta, KatsumiBielen, AnaMlynarczyk-Bonikowska, BeataBierza, WojciechMo, Solveig SølverødBierzuńska, PaulinaMoawad, AmiraBiessy, AdrienModenese, AlbertoBilli, DanielaMoens, Ugo LionelBilski, JanMohamed, Ashour FikryBinati, Renato L.Mohamed, El-SaadonyBinder, LinaMohamed, HassanBingham, RichardMohamed, Mahmoud S. M.Bîrluţiu, Rareş MirceaMohammad, MobashirBirsa, Mihail LucianMohammed, RababBjelic, DraganaMohsin, MuhammadBlacha-Grzechnik, AgataMojicevic, MarijaBlaga, Alexandra CristinaMoleleki, LucyBlaha, ThomasMolina, IsraelBlanco, JorgeMolina, LázaroBlank, RalfMolina-Guerrero, Carlos EduardoBlanton, LucasMolino, PaulBlasdell, KimMoloney, Gerard M.Blasi, ElisabettaMonaco, FedericaBłaszczyk, LidiaMonchatre-Leroy, ElodieBlauth de Lima, FernandaMoniello, GiuseppeBlawid, RosanaMonro, JohnBlazhev, AlexanderMonroy, FernandoBleotu, CoraliaMonteiro, SílviaBlesa, AlbaMonteiro-Neto, ValérioBlifernez-Klassen, OlgaMontemurro, MarcoBlondeau, JosephMontforte, Antonella D’ArminioBoattini, MatteoMontiel, JesúsBobis, OtiliaMontoro, MiguelBoccia, GiovanniMontso, Peter KotsoanaBochniarz, MariolaMonzote, LianetBody-Malapel, MathildeMookherjee, AbhirupBoerlage, AnnetteMora, Delio JoséBogiel, TomaszMorales, DiegoBohacz, JustynaMorales-Luckie, Raúl AlbertoBohmwald, KarenMora-Montes, Hector M.Bojarski, BartoszMoran, JamesBok, EwaMorar, AdrianaBonam, Srinivasa ReddyMoreira, Irina S.Bonciu, ElenaMoreira, MárcioBoncompagni, EricMorello, WilliamBonetta, SaraMoreno Mesonero, LauraBonetta, SilviaMoreno, Andrea MickeBonilauri, PaoloMoreno-Garrido, IgnacioBonilla, DeniseMoretti, SoniaBono, JamesMorfin, NuriaBorba Dos Santos, Luana PereiraMorgan, NoelBorges, ClarissaMorgunov, IgorBorges, SandraMori, EmilianoBorkiewicz, LidiaMorita, YukioBorruso, LuigimariaMoriya, HisaoBorszewska-Kornacka, MariaMorley, KristaBorza, TudorMorozov, Sergey Yu.Bose, ChhandaMorozova, OlgaBotelho, AnaMorris, JamesBotelho, PatríciaMorrison, Lynda A.Bouchami, OnsMorse, DavidBoudin, HélèneMoşneguţu, Emilian FlorinBouizgarne, BrahimMoss, Elica M.Bourgeois, DenisMossialos, DimitrisBourret, TylerMott, AlexanderBoussaa, SamiaMotta, Jean-PaulBowden, TimothyMottran, JeremyBowman, John P.Moula, NassimBozdoğan, HakanMsimang, VeerleBozzo, GiancarloMu, Bo-ZhongBranchu, PriscillaMuders, ThomasBransfield, RobertMuggeo, PaolaBrasel, TrevorMukhanov, Vladimir S.Braun, BurgaMullan, MichaelBrayton, KellyMüller, JoachimBreitenbach, MichaelMüller, Jochen A.Brener, BeatrizMüller, ThomasBreyton, CécileMullié, CatherineBriandet, RomainMullineaux, Conrad W.Brígido, ClarisseMunch, Jean CharlesBrin, MitchellMunir, ShahzadBrinkmann, HennerMuñoz-Antoli, CarlaBrisabois, AnneMuntean, Alexandru AndreiBrisse, MorganMuntyan, Maria S.Brito, LuísaMuraki, YuichiBrito, MiguelMuramatsu, YasukazuBrito, Nuno V.Murata, KazumotoBritz, Margaret L.Murillo, JesusBrizić, IlijaMurphy, Eain A.Brocchi, MarceloMurphy, ShonaBrogi, SimoneMusatti, AlidaBrown, LindsayMustafa, AdnanBrözel, VolkerMuszyński, SiemowitBrugère-Picoux, JeanneMutalipassi, MirkoBrüggemann, YannickMuto, YoshinoriBrunetto, Marcio AntonioMuyyarikkandy, Muhammed ShafeekhBruno, FedericaNa, Byoung-KukBruno, GiuseppeNaama, Lang-YonaBrycki, BogumilNaber, KurtBrygadyrenko, Viktor V.Nagao, Prescilla EmyBrzozowska, EwaNagarajan, DilliraniBuan, NicoleNagy, TiborBubak, IwonaNai, Yu-ShinBuchovec, IrinaNakagawa, TomoyukiBuckley, AlexandraNakanishi, NorikoBudaeva, Vera V.Nakayama, YoshitakaBudisa, NediljkoNale, JanetBudryn, GrażynaNapiórkowska-Krzebietke, AgnieszkaBudzynska, SylwiaNapoli, ChristianBueno De Mesquita, Clifton P.Napoli, EttoreBugarin, AlejandroNarayan, Om PrakashBugelli, ValentinaNarra, Hema PrasadBuitrago, María JoséNasher, FauzyBukauskaitė, DovilėNasir, FahadBulaev, AleksandrNasr, MahmoudBulatov, EmilNastasa, CristinaBulgariu, LauraNastasa, ValentinBumunang, EmmanuelNastuta, Andrei VasileBuonomo, Antonio RiccardoNatale, GianfrancoBurge, Kathryn Y.Natarajan, PurushothamanBurgess, GrahamNatekar, Janhavi P.Burgos, MorenoNath, ArijitBurián, KatalinNathanailides, CosmasBurlakovs, JurisNatu, RuchaBurnap, RobNaughton, PatrickBuscemi, Maria DrussillaNavaratnarajah, ChanakhaBustos-Terrones, YanethNavarro-Torre, SalvadoraButcher, JamesNavas, AlfonsoButenko, AnzhelikaNavas, JesúsButiuc-Keul, Anca LiviaNavascues, EvaButts, ChrissieNaydenov, Nayden G.Cabezas-Cruz, AlejandroNayla, MunawarCaboň, MiroslavNazik, HasanCadaret, CaitlinNazli, AishaCadel-Six, SabrinaNazzaro, FilomenaCadillo-Quiroz, HinsbyNeagu, SimonaCaetano, TaniaNędzarek, ArkadiuszCafiso, AlessandraNeelakantan, PrasannaCai, YiminNegi, ArvindCaito, SamuelNeri, IriaCalcutt, MichaelNesler, AndreaCalderón, Iván L.Neto, HelenaCalina, DanielaNevárez-Moorillón, Guadalupe VirginiaCaljon, GuyNeves, FelipeCalla, JaesonNguyen, Le PhuongCallahan, Brian P.Nguyen, TrieuCallanan, MichaelNguyen, Van KhanhCallegari, Maria LuisaNicholas, RobinCaller, LauraNicholson, Ainsley C.Calvert, Amanda E.Nicolas, Francisco E.Calvete, MarioNicolas, MassartCalvo, Maria AngelsNicoletti, RosarioCamacho, Irene Gomes CâmaraNicosia, AldoCamacho-Ruiz, Rosa MaríaNiculae, MihaelaCamp, Jeremy V.Nie, YongCampanale, ClaudiaNielsen, Vance G.Campello, PaulaNieminen, PenttiCampera, MarcoNiestępski, SebastianCampo, Rosa DelNiewiadomska, AlicjaCampos, CatarinaNigam, DeeptiCampos, Joaquín MaríaNigris, SebastianoCampos, Maria Jorge GeraldesNigro, RobertoCampos, Milton Cesar CostaNikitin, Dmitry А.Canchi, SaranyaNikolaeva-Glomb, LubomiraCandido, SaverioNikolausz, MarcellCanuti, MartaNikolova, KrastenaCaparros-Martin, JoseNikolova, MariaCapela e Silva, FernandoNikouli, EleniCapela, DelphineNishida, TakehiroCapelli-Peixoto, JanaínaNishide, YudaiCaponio, FrancescoNishino, NaokiCapra, Gian FrancoNisnevitch, MarinaCaprari, ClaudioNissan, IsraelCaputo, LeonardoNixon, PeterCarareto Alves, Lucia MariaNizyaeva, Natalia ViktorovnaCarbone, Dora AllegraNjage, PatrickCarbonell, PabloNocera, Francesca PaolaCardinali, GianluigiNoda, MasafumiCardoso, LuísNogueda-Torres, BenjaminCardoso, PauloNogueira, TeresaCardoso, Tereza C.Noiri, YuichiroCarmignani, MarcoNora, Flávia DallaCarneiro, Lilian CarlaNoreen, ZobiaCarneiro, Sueli Coelho Da SilvaNoreldin, AhmedCarnell, GeorgeNörz, Dominik SebastianCarnevale Miino, MarcoNotarte, Kin IsraelCarradori, SimoneNovais, CarlaCarranca, CorinaNowakowski, MichałCarrasco, Carlos J.Ntaikou, IoannaCarrasco, JaimeNunes, Maria AntoniaCarretto, EdoardoNurjadi, DennisCarrino-Kyker, Sarah R.Nürnberg, DennisCarroll-Portillo, AmandaNuttall, PatCarter, KatharineNyaga, MartinCartmill, Andrew D.Nzila, AlexisCarvalho, EugeniaO’Brien, Caitlin A.Casado-Bedmar, MaiteOakley, AaronCaserta, SergioObama, TakashiCasino, PatriciaO’Callaghan, DavidCassier-Chauvat, CorinneOchowiak, MarekCastell Escuer, MargaridaO’Connor, RobertaCastelli, GermanoOdaka, YoshinobuCastro-Longoria, ErnestinaOdamaki, ToshitakaCaswell, Clayton C.Oduor, Joseph Michael Ochieng’Catania, ValentinaOem, Jae-KuCattoni, FrancescaOgawa, AkikoCayol, Jean-lucOgino, ShujiCeballos, GuillermoOgorek, RafalCeballos, Nidia AréchigaOgórek, RafałCebrián Castillo, RubénOgrodowczyk, AnnaCebulak, TomaszOh, Seung-YoonCeci, AndreaO’Halloran, ConorCecilia, HodurÖhrfelt, AnnikaCelandroni, FrancescoOhtomo, RyoCeniti, CarlottaOhyama, TakujiCennamo, PaolaOishi, TomohiroCerbo, Alessandro DiOkamoto, HiroakiČernáková, LuciaOkińczyc, PiotrČerný, JiříOkorski, AdamCerqueda-García, DanielOksenych, ValentynCerri, MartinaOkumura, KoCerutti, AndreaOlalde-Portugal, VictorĆetković, GordanaOld, JulieCevallos, Miguel A.Ołdziej, StanislawChajȩcka-Wierzchowska, WioletaOleastro, MónicaChalvon-Demersay, TristanOlejniczak, IzabellaChan, MartinOlejnik-Schmidt, AgnieszkaChan, SunneyOleskin, Alexander V.Chandrasekaran, MurugesanOlga, PetrovaChang, JinhongOliva, GaetanoChang, Qiao-ChengOlivares, FabioChao, Chien-MingOliveira, LauraChapartegui-González, ItziarOliveira, ManuelaChapman, Nora M.Oliveira, Marcia Cristina De SenaCharpentier, ElénaOlivera, Elías R.Chatzigiannidou, IoannaOlszewski, Michal AChatzipanagiotou, StylianosOmaña-Molina, MaritzaChatzis, LoukasOndreičková, KatarínaChatzopoulos, Dimitris C.Ong, EdisonChaudhary, Dhiraj KumarOni, Feyisara EyiwumiChaudhuri, MinuOnodera, TakashiChávez-Calvillo, GabrielaOnyedibe, Kenneth I.Chebotar, VladimirOp Den Camp, H. J. M.Chedea, Veronica SandaOpavski, NatasaChekanov, KonstantinOprea, Ovidiu CristianChen, Chien-HungOrasch, ThomasChen, ChuanOrefice, IdaChen, Chyi-LiangOrellana-Palma, PatricioChen, FengjuiOresnik, IvanChen, Jiann-ChuOrfanoudakis, MichailChen, Jia-YuhOrłowska, BlankaChen, Jin-PingOrosz, FerencChen, Jung-ShengOroz-Guinea, IsabelChen, MinOrsi, EnricoChen, PengOrsini, MassimilianoChen, ShaohuaOrtega, JosephChen, TingtaoOrtega, María JesúsChen, WanhaoOrtega, Miguel A.Chen, XaolinOrtega-santos, CarmenChen, YaoxingOrtiz-Andrade, RolffyChen, Yi-HsingOrtuño, MariaChen, Yuan-ChuanOrusa, RiccardoChen, Yuh-shuenOrzechowski Xavier, MelissaChen, ZunweiOsajima, Josy AnteveliCheng, HengweiOsaka, ToshifumiCheng, JinhuaOscorbin, IgorCheng, Kuan-ChenOsińska, AdrianaCheng, LiangOsman, MarwanCheng, Yeong-HsiangOsowiecka, KarolinaChenoll, EmparO’Sullivan, JefferyChernitsyna, Svetlana M.Osuna, AntonioChesdachai, SupavitOszako, TomaszChetta, MassimilianoOtani, NorioChetverikov, SergeyOtelea, DanChevalier, FredericOtu, HasanChi, Chih-YuOtulak, KatarzynaChiang, Chih-PoOviedo-Silva, Claudia A.Chiang, Yin-RuOzga, AndrewChiang, Yu-ChungOzoline, OlgaChiang-Ni, ChuanPace, LorenzoChiarelli, LaurentPacholak, AmandaChifiriuc, CarmenPacífico, CátiaCho, KyungjinPacioni, GiovanniCho, Won KyongPadilla, DanielChochlakis, DimosthenisPadkina, MarinaChoe, UyoryPadkina, Marina V.Choi, Jong SooPadrão, JorgeChoi, Yong-KeunPadrós, FrancescCholewińska, PaulinaPaduch, RomanChoque, ElodiePagliarulo, CaterinaChow, Franklin Wang-NgaiPagotto, Franco J.Chow, Yen-HungPagou, ΚalliopiChowdhury, SouravPai, Tzu–YiChristie, GrahamPaine, KevinChristina Pillon, MonicaPajdak-Czaus, JoannaChristodoulides, MyronPak, Sok CheonChrysanthakopoulos, Nikolaos A.Pakbin, BabakChu, HongminPalacios, José-ManuelChu, Luan LuongPaleczny, JustynaChu, ZhaohuiPalma, Paulo J.Chua, Eng GuanPalomar, Ana MariaChung, ChihchingPalomba, MarialetiziaChung, JasminePaluch, EmilChung, Wen-HsinPalusinska-Szysz, MartaChung, Won-HyongPanaro, Maria AntoniettaChwastowski, JarosławPandíțh, AnúpCiancio, AurelioPanebianco, FeliceCiani, MaurizioPang, BoCiardiello, Maria AntoniettaPanikov, NicolaiCichero, ElenaPanke-Buisse, KevinCichosz, MarcinPanov, AlexeyCiesielski, SlawomirPantalone, Mattia RusselCieslak, AdamPanthee, SureshCieslar, GrzegorzPaolucci, MarinaCinco, MarinaPapadimitriou, KonstantinosCipak, LubosPapadopoulos, Antonios N.Citu, CosminPapadopoulos, Elias G.Ciulli, SaraPapadopoulos, TheofilosCiura, KrzesimirPapaianni, MarinaClay, KeithPapatheodorou, EfiCluck, DavidPapatsiros, VasileiosCoates, HarveyPappa, Olga D.Cocchi, MoniaPappas, Christopher J.Cochis, AndreaParamythiotis, SpirosCoelho, Ana CláudiaParanhos-Baccalà, GlauciaCohen, RegevParanthaman, Balasubramani SubramaniColitti, BarbaraPardini Gontijo, Marco TúlioColonna, BiancaPardo, Juan PabloColussi, SilviaParedes-Esquivel, Claudia CaterinaComas-Garcia, AndreuParis, LucCombrink, LeighPark, AelyCompain, FabricePark, Chang EonConstantin, Oana EmiliaPark, GyungsoonContaldo, NicolettaPark, Hee-MoonContreras, AsunciónPark, HyunCoppi, MarcoPark, Jae YongCordaro, MassimoPark, Jong MyongCordero-Bueso, GustavoPark, Seong-JunCordero-Otero, Ricardo R.Park, Sun YoungCorreia, MariaPark, Yeong-JunCorretto, ErikaParker, CraigCortés-Hinojosa, GalaxiaParlapani, Foteini F.Cortes-Perez, Naima G.Parola, PhilippeCosby, Douglas E.Parra-Flores, JulioCosby, Sara LouiseParreira, RicardoCosma, PinalysaParrino, VincenzoCosoveanu, AndreeaParuch, LisaCosseddu, Gian MarioParvanova-Mancheva, TsvetomilaCossio-Bayugar, RaquelPascadopoli, MaurizioCosta, DamienPaściak, MariolaCosta, Diogo Fleury AzevedoPasechnik, TatyanaCosta, EduardoPashkovskiy, PavelCosta, Ohana Y. A.Passera, AlessandroCostantini, AntonellaPassero, Luiz Felipe D.Cotârleț, MihaelaPastirčáková, KatarínaCoufal, StepanPastor, VictoriaCoutinho, TeresaPastore, Glaucia MariaCozzani, EmanuelePastorino, PaoloCraina, MariusPatchett, MarkCrandall, PhilipPatel, Anil K.Crecchio, CarminePatel, GopalCrisan-Dabija, Radu AdrianPatel, Jai SinghCristina, Romeo T.Patel, SanjayCrnkovic, AnaPatenge, NadjaCroft, LarryPathania, Anup S.Crook, BrianPatkowska, ElżbietaCruz, CristinaPatra, Jayanta KumarCruz, JoanaPatterson, MurrayCubero, JaimePatuzzi, IlariaCucco, MarcoPatz, SaschaCui, FengchaoPaul, JoydeepCui, HaiyangPaul, Narayan ChandraCunha, EvaPaula, Vanessa BrancoCunningham-Oakes, EdwardPaulette, LauraCuozzo, Sergio AntonioPaulino-Lima, Ivan GláucioCuria, Maria CristinaPaulo Vicente, Ana CarolinaCurutiu, CarmenPaungfoo-Lonhienne, ChanyaratCwiklinski, KrystynaPavelka, Martin S.Cycoń, MariuszPavli, FoteiniCyplik, PawełPavloudi, ChristinaCzaja, KrzysztofPavlov, Igor N.Czerniejewski, PrzemysławPavlović, HrvojeCzerwik-Marcinkowska, JoannaPavoni, MatteoDa Costa, José Manuel CorreiaPawlik, AnnaDa Cruz, Georgiana F.Pawlikowska-Pawlęga, BożenaDa Rold, GrazianaPawłowska, MałgorzataDa Rosa, Joao AristeuPaz Saavedra, ClaudiaDa Silva Vale, AlexanderPech-Cervantes, Andres AlfredoDa Silva, Luís C. N.Pecher, Wolf T.Dabert, MirosławaPecka-Kielb, EwaDąbrowska, JoannaPedelacq, Jean-DenisDąbrowski, WojciechPederiva, SabinaDaddam, Jayasimha RayaluPedroso, Reginaldo Dos SantosDahlgren, DavidPei, HaiyanDaly, Seth M.Pekala-Safinska, AgnieszkaDame, RemusPekmezovic, MarinaD’Amico, FedericaPellegrini, MarikaDandekar, ThomasPellegrini, Massimiliano MatteoDanilenko, Daria M.Pellicano, RinaldoDaria, MatyushkinaPeneva, VladaDarveau, RichardPeng, CongyueDasiewicz, KrzysztofPeng, DapengDatta, RahulPensec, FloraDavid Vejarano Mantilla, RicardoPepe, TizianaDavid, Michael Z.Pepi, MilvaDavid, PiresPeralta-Ruiz, YeimmyDavolos, DomenicoPereira, Ana MargaridaDavydenko, KaterynaPereira, Carlos DiasDe Almeida, José RafaelPereira, FilipaDe Almeida, RoneiPereira, Marcelo MaiaDe Alteriis, ElisabettaPerelomov, Leonid V.De Andrade, Cristiano J.Peretz, AviDe Angelis, BarbaraPérez-Sánchez, RicardoDe Bellis, MirellaPérez-Santos, MartínDe Brum Vieira, PatríciaPérez-Tanoira, RamónDe Castro Ramalho, TeodoricoPerfetto, BrunellaDe Chiara, GiovannaPerner, JanDe Cock, HansPerreault, JonathanDe Craene, Johan-OwenPerry, John D.De Curtis, FilippoPertile, GiorgiaDe Felice, ElenaPeruzy, Maria FrancescaDe Giglio, ElviraPerveen, NighatDe Giglio, OsvaldaPessela João, BenevidesDe Gregorio, ElianaPessi, TanjaDe Jesus Inácio Belas, AdrianaPeszek, ŁukaszDe La Cruz, Armah A.Peta, VincentDe La Torre, BasilioPetca, AidaDe Lana, MartaPetersen, JörnDe Luca, DanielePetinaki, EfthimiaDe Lurdes Dapkevicius, MariaPetkova, MarianaDe Melo, Angelita CristinePetrarca, LauraDe Oliveira, Tadeu SilvaPetri, Renee M.De Simone, Salvatore GiovanniPetronio Petronio, GiulioDea-Ayuela, MaríaPetronio, GiulioDearborn, Altaira D.Petrova, MayyaDeb, SubrataPetrova, SlaveyaDec, MartaPetryszak, PrzemyslawDedicoat, Martin JohnPhilippe, CécileDeere, JessePhillips, Kenneth ScottDefilippo, FrancescoPiatek, JacekDeghmane, Ala-EddinePichon, MaximeDegiuseppe, JuanPickett, AndyDegli Esposti, MauroPiecuch, AgataDegola, FrancescaPiekarska, KatarzynaDel Gallo, MaddalenaPiergiacomo, PiergiacomoDelannoy, SabinePiestansky, JurajDelbue, SerenaPieszka, MarekDeledda, AndreaPietragalla, MicheleDeligianni, ElenaPietrangelo, LauraDelmont, EmilienPignataro, GiuliaDemetrescu, IoanaPilarska, Agnieszka A.D’Emilio, MariagraziaPilloni, GiovanniDeng, LeiPimenov, NikolaiDeng, LonghuiPineda-Hidalgo, Karen V.Deng, YalePinedo-Rivilla, CristinaDeng, YijiePinheiro, Roberta OlmoDenorme, FrederikPinkas, AdiDepa, ŁukaszPinsino, AnnalisaD’Ercole, SimonettaPinto, GraçaDeryabina, Yulia I.Pinzari, FlaviaDeshmukh, RupeshPiotrowska-Cyplik, AgnieszkaDesnos-Ollivier, MariePiper, Peter W.Dessau, RamPircalabioru, Gratiela GradisteanuDesselberger, UlrichPires Hardoim, Cristiane CassiolatoDeviatkin, Andrei A.Pirhonen, MinnaDevkota, Hari PrasadPironti, ConcettaDévora-Isiordia, Germán EduardoPissetti, CarolineDey, DebajitPistorio, ValeriaDhakal, NirajanPituch, Hanna M.Dhakal, SantoshPiwowarek, KamilDhawi, FatenPlante, Craig J.Di Cosola, MichelePlata-Rueda, AngelicaDi Donato, PaolaPlattner, HelmutDi Filippo, MarziaPławińska-Czarnak, JoannaDi Gennaro, FrancescoPleckaityte, MildaDi Giallonardo, FrancescaPleskova, SvetlanaDi Giovanni, PamelaPlessas, StavrosDi Marco, RobertoPłoszaj, TomaszDiakou, AnastasiaPlotnikov, AndreyDias, André AlvesPobiega, KatarzynaDias, TeresaPobiega, MonikaDiaz Fernadez, PabloPodleśny, JanuszDíaz-López, MartaPoetsch, AnsgarDíaz-Sánchez, Adrian AlbertoPoitevin, Carolina G.Dickenson, Nicholas E.Pokrovsky, Oleg S.Dickey, AaronPoli, AnnaritaDickson, BrentPolicastro, Steven A.Diez-Quijada, LeticiaPolimeno, LorenzoDileepan, MythiliPolivtseva, Valentina N.Dilfuza, EgamberdievaPoluéktova, Elena U.Dimitrellou, DimitraPomel, SebastienDimitrov, DimitarPomilio, FrancescoDimov, IvanPongor, SandorDimova, IvankaPoniatowski, Łukasz A.Din, Ahmad UdPonte-Sucre, AliciaDinca, LucianPop, Lecturer Carmen RodicaDinleyici, EnerPop, Tudor LucianDinte, ElenaPop, Tudor SorinDiPalma, GiannaPopa, Gabriela Loan LoredanaDireito, RosaPopa, Ioan MirceaDiRuggiero, JocelynePopa, Iolanda ValentinaDiţu, Lia MaraPopa, Mircea IoanDjordjevic, SnezanaPopescu, Corneliu PetruDjordjevic, Steven. P.Popescu, Cristina Raluca Gh.Długaszewska, JolantaPopova, Alexandra A.Doak, Thomas G.Popova, BlagovestaDobay, OrsolyaPopović, NikolaDobrovolny, Hana M.Popovici, VioletaDobrowolski, PiotrPoppers, DavidDobrzański, Lech B.Portillo López, AmeliaDocea, AncaPortugal, AntonioDodds, W. JeanPoulin, LucieDoherty, Jean FrançoisPozos-Guillén, AmauryDolfing, JanPozzobon, VictorDomann, EugenPrakas, PetrasDomenzain, IvánPrats, María SoledadDonadu, MatthewPrazdnova, Evgeniya Valer EvnaDong, QingliPrearo, MarinoDonovan, NeridaPresentato, AlessandroDonta, SamuelPrete, RobertaDorantes-Aranda, Juan JoséPrete, Sonia DelDorcioman, GabrielaPretto, TobiaDoroftei, BogdanPrice, PatriciaDos Santos, Helcio SilvaPrieto, HumbertoDos Santos, Ricardo FilipePrimon-Barros, MurielDos Santos, Ronaldo Vagner ThomatieliPrivitera, GaetanoDos Santos, Tatiana CarlescoPriyadarshini, MedhaDrago, LorenzoProbanza, AgustinDrahovská, HanaProctor, Caitlin R.Driessen, Arnold J. M.Prodhan, Zakaria HossainDrillich, MarcPronost, StephaneDruszczynska, MagdalenaPrpic, JelenaDu, DongyunPrpić, ZvonimirDu, RenpengPrudnikova, S. V.Du, WeiPrykhodko, OlenaDuan, XuefengPrywer, JolantaDuanis-Assaf, DaniellePrzemieniecki, Sebastian WojciechDubey, Ashutosh P.Psaroulaki, AnnaDubey, Jitender P.Ptaszek, AnnaDucatez, MariettePu, MengDuda-Chodak, AleksandraPucciarelli, SandraDuda-Madej, AnnaPuglia, Anna MariaDufossé, LaurentPugliese, MichelaDulf, FranciscPuopolo, GerardoDumancas, GerardPurkrtová, SabinaDumenyo, KorsiPutrinš, MartaDumic, IgorPyzik, AdamDumitru, Irina MagdalenaPyz-Łukasik, RenataDunaj, JustynaQader, M. MalliqueDunisławska, AleksandraQi, WeihongDurán-Riveroll, LorenaQian, XiaoqingDurigan, MauricioQuatrin, Priscilla MacielDutta, NalokQuelemes, PatrickDutta, SanjuctaQuéméneur, MarianneDvoretsky, Alexander G.Quesada Pérez, VíctorDvoretsky, VladimirQuiñones, BeatrizDvorožňáková, EmíliaQuiroz-Castañeda, Rosa EstelaDykes, Gary A. A.Rabah, HouemDylag, MariuszRadcliff, Fiona JaneDzhalilov, Fevzi S.-U.Radić, TomislavDziekiewicz, MarcinRadu, Nicoleta NicoleDzimianski, JohnRahman, AdeebEdwards, Adrianne N.Rahman, AzizurEfremenko, ElenaRahnavard, GholamaliEfremenkova, Olga V.Raj Puri, RameshEfthimiou, GeorgeRajasekharan, Satish KumarEgamberdieva, DilfuzaRajput, VishnuEhling-Schulz, MonikaRajter, LubomirEhmann, RosinaRakov, Alexey V.Ehrhardt, ChristopherRamalingam, SrinivasanEisenreich, WolfgangRamasamy, RanjanEkateriniadou, LoukiaRamilo, DavidEkonomou, SotiriosRamírez, Ana SofíaEl Barnossi, AzeddinRamirez, Jose LuisEl-Adawy, HosnyRamos López, Miguel ÁngelElbestawy, AhmedRamos-Rodríguez, Juan JoséElfeil, WaelRampazzo, Rita De Cássia PontelloEl-Ghany, Wafaa A. AbdRamsay, JulianaElie, Desmond-Le QuemenerRana, JyotiEl-Kholy, Amani AliRanadheera, SenakaElkins, KellyRanitović, AleksandraEllepola, KassapaRanjan, AnujEllis, Keith C.Ranjan, KishuEllwanger, JoelRapoport, AlexanderElmegharbel, Samy MahmoudRaposo, RosaEl-Sawah, AhmedRašić, DubravkaElshafie, Hazem S.Rasiukevičiūtė, NeringaElsohaby, IbrahimRasmussen, Torben SølbeckEltanahy, EladlRastelli, EugenioEl-Tarabily, KhaledRathod, JagatElumalai, PunniyakottiRathod, SanjayEnache, Mădălin-IancuRatiu, AndreeaEnany, ShymaaRauch, JessicaEngesser, Karl-HeinrichRavagnan, SilviaEngevik, Melinda A.Raven, John AlbertErgönül, ÖnderRe, SuyongEriani, GilbertReale, StefanoEricson, Marna E.Redd, Travis K.Ermolenko, ElenaReginatto, ValeriaErol, İrfanRehfeld, Jens FrederikEscario, JoséReich, AdamEsneau, CamilleReichel, Michael P.Espenberg, MikkReid, Cameron J.Espinosa, María Del Rosario MoralesReis, RicardoEssock-Burns, TaraRelllegadla, SandeepEsteban, JaimeRementeria, AitorEsteves, EduardoRemesar, SusanaEstrada-Peña, AgustínRen, LijuanEtchegarray, AugustoRenault, PierreÉva, MikóRequena, Jose MaríaEvangelista, DominicResch, BernhardEvans, Alyssa B.Reuter, KlausEvsyutina, Daria V.Reva, OlegExindari, MariaRey, LuisFacey, PaulReyes, FernandoFafutis-Morris, MaryReyes-Batlle, MaríaFagen, JennieReyes-Vidal, YolandaFagoonee, SharmilaRibeiro, ThierryFagorzi, CamillaRicca, EzioFalagán, CarmenRicci, AnnalisaFaleiro, Maria LeonorRichard, VincentFalkinham, Joseph O.Richardson, ElisabethFally, MarkusRichert, AgnieszkaFancello, FrancescoRichi, PatriciaFang, XuanRichter, PeterFani, RenatoRidley, AnneFantacuzzi, MarialuigiaRiehle, MichaelFarabaugh, Philip JamesRijpkema, SjoerdFarcasanu, Ileana C.Rimsky, SylvieFardeau, Marie-LaureRinder, MonikaFaré, Pietro BenedettoRío, Pablo G. DelFarkas, Edit É.Riool, MartijnFaron, Matthew L.Rios-Velasco, ClaudioFarone, Mary B.Ripabelli, GiancarloFarrer, RhysRissmann, MelanieFarsang, AttilaRivas-Marin, ElenaFasanella, AntonioRivera, DácilFasciana, TeresaRivero-Pérez, Maria DoloresFazzio, Luis EmilioRivero-Perez, NallelyFederici, MassimoRizo-Liendo, AitorFederici, SaraRobak, MałgorzataFelgueiras, HelenaRoberts, Sigrid C.Feliciano, JoanaRobinson, KelsyFendrihan, SergiuRocha Faustino, Ana IsabelFeng, Cui-PingRodamilans, BernardoFeng, HonglinRodrigo, ChaturakaFeng, JiaoRodrigues, ArmandaFeng, NingguoRodrigues, Clara F.Feng, ZongdiRodrigues, Yan CorrêaFenton, Heather M. A.Rodríguez, AlexisFerdeș, MarianaRodríguez-Expósito, Rubén L.Ferdous, JinnatRodríguez-Iglesias, Manuel A.Fernandes, JorlanRodriguez-Llorente, Ignacio D.Fernandes, Maria C.Rodríguez-López, María IsabelFernandez, Ana IsabelRodríguez-López, PedroFernández-Arévalo, AnnaRodríguez-Negrete, EdgarFernández-Coll, LlorençRodríguez-Noriega, EduardoFernández-Figueroa, Edith A.Roesch, FerdinandFernández-Tomé, SamuelRojas-Lopez, MaricarmenFerrara, ElisabettaRojo, Maria AngelesFerrara, FrancescoRokosz, KrzysztofFerrara, GianmarcoRolbiecki, DamianFerraz, Maria PiaRollini, ManuelaFerreira, Ana CristinaRoman, KamilFerreira, Carlos Miguel HenriquesRomano, ElioFerreira, HenriqueRomano, GiovannaFerreira, Manuel MarquesRomano, IdaFerreira, PedroRomano, PatriziaFerreira-Paim, KennioRomantschuk, MartinFerreira-Santos, PedroRomero Noguera, JulioFiedler, TomasRomero, Jaime MoisésFigueiral, Maria HelenaRomo, MariaFigueiredo, ElisabeteRon, ElioraFigueroa, John PeterRong, Chan KuanFigueroa-Millán, Julio VicenteRosales, RubenFilardo, SimoneRosana, Albert Remus R.Filipič, BratkoRosas, IrmaFink, RokRosberg, Anna KarinFinore, IlariaRoscetto, EmanuelaFiorenza, SalvatoreRoselle, Gary A.Fischer, Egil Andreas JoorRosenthal, BenjaminFlaherty, RebeccaRosito, MariaFlint, SteveRossez, YannickFlorentino, Anna PatrícyaRossi, FrancaFlorescu, Simin-AyselRoszek, KatarzynaFlorín-Christensen, MonicaRouers, AngelineFokin, Sergei I.Rouge, PierreFöldvári, GáborRoumiantseva, Marina LvovnaFoley, SophieRouphael, YoussefFonseca, KevinRoussis, IoannisFontana, CarlaRoy, AmitFort, IsabelRóżańska, AnnaFoster, DerekRozhina, ElviraFoster, TimRubio, LuisFothergill, JoanneRudi, LudmilaFournier, GregoryRudoy, DmitriyFox, Eduardo Gonçalves PatersonRueda-Ruzafa, LolaFraga, MartínRuelland, EricFranceschi, PieroRuffin, FeliciaFranchi, ElisabettaRuiz, EnricoFranciosi, ElenaRuiz, FacundoFranciosini, Maria PiaRuiz-Lau, NancyFranciotti, RaffaellaRump, KatharinaFranco-Duarte, RicardoRupp, SteffenFrancois, PatriceRusenova, Nikolina VelizarovaFrangeul, LionelRusic, DorisFranzolin, Marcia ReginaRusu, AlexandruFrasca, FedericaRusu, TeodorFraser, JohannaRuta, LaviniaFratini, FilippoRuzek, DanielFrean, JohnRyabchikova, Elena I.Freddi, LucaRyan, FrankFreestone, PrimroseRychert, KrzysztofFremin, Brayon J.Rymaszewska, AnnaFrías-De-León, María GuadalupeRysiak, AnnaFrickmann, HagenRząd, IzabellaFrietze, Seth E.Sa, TongminFrillingos, StathisSaad, Ahmed MohamedFrišták, VladimírSaar Gomes, RodrigoFritz, InesSabaneyeva, Elena V.Frutos Pérez-Surio, AlbertoSabino, RaquelFrutos, María CeliaSachivkina, NadezhdaFrydas, IliasSacks, David LawrenceFrye, JonathanSadeghianmaryan, AliFuentes, Màrius V.Sadeghifar, HasanFujita, MasakiSadlova, JovanaFukuda, YasuhiroSafonov, AlexeyFukushima, KentaroSaggu, GagandeepFurlanetto Pacheco, Ana BeatrizSaha, SudeshnaFurmanczyk, EwaSaingam, PrakitFurtado, Edson LuizSaito, JumpeiFurtak, KarolinaSaiz Jimenez, CesareoFurukawa, KenjiSajid, MuhhamadGabrić, DraganaSak, Jarosław J.Gabriel, IwonaSakellis, EliasGajdács, MárióSalamon, DominikaGajewska, JoannaSalek, KarinaGalabov, AngelŠalić, AnitaGalachyants, Agnia D.Salmanton-García, JonGalaitsi, Stephanie E.Salmeri, MarioGalan, Ana-MariaSalzano, FrancescoGalanis, AlexisSamac, Deborah A.Galante, DomenicoSamardžija, MarkoGaldiero, EmiliaSamarzija, IvanaGałęcki, RemigiuszSambri, AndreaGaletto, LucianaSamelis, JohnGallegos, AlbertoSampaio, AnaGallelli, LucaSamrat, Subodh K.Gallert, ClaudiaSamylina, Olga S.Gallier, SophieSanchez, SusanGallo, GiovanniSanchez-Amat, AntonioGallo, MariannaSanchez-Betancourt, JoseGallo, OresteSandle, TimGalluzzo, PaolaSándor, Attila D.Gama, JoaoSándor, ErzsébetGamalero, ElisaSandoval-Sierra, Jose VladimirGambino, Caterina MariaSanjuán, JuanGamez, GustavoSanmukh, Swapnil GaneshGamez, StephanieSantacroce, LuigiGan, JinhuaSantaella, MarinaGandla, Madhavi LathaSantana, Andrés GonzálezGanesan, A.Santander, JavierGanesan, RajaSantaniello, AntonioGangcuangco, Louie Mar A.Santarem, NunoGanz, Holly H.Šantek, Mirela IvančićGao, BeileSantella, BiagioGao, MengjiaoSantiago, Araceli E.Gao, QiangSantiago-Rodriguez, Tasha MarieGao, YangSantini, AntonelloGarbacz, KatarzynaSantos, Jesús A.Garbayo, InésSantos, MilaGarcia, MiriamSantos-López, GerardoGarcía, VerónicaSantovito, GianfrancoGarcía-Carretero, RafaelSantoyo, GustavoGarcia-Longoria, LuzSar, TanerGarcía-R, Juan CarlosSaraiva, MarciaGarcía-Ríos, EstéfaniSaratale, Ganesh DattatrayaGarcovich, DanieleSardo, AngelaGardner, Jennie M.Šaric̈, LjubišaGarduño, Rafael A.Sarkar, SoumyadevGargala, GillesSarshar, MeysamGargari, GiorgioSartini, MarinaGarnaud, CecileSartor, AssuntaGarofolo, GiulianoSathiyaraj, SrinivasanGarrido, VictoriaSato, HiroshiGarrido-Sanz, DanielSato, TakashiGasmi, LeilaSato, TakuichiGaspar, CarlosSavvidou, MariaGasparatos, DionisiosSawicka, BarbaraGaspersic, RokSayed, Ibrahim M.Gaurivaud, PatriceSazonova, Margarita A.Gautam, SaurabhScamporrino, Andrea AntoninoGauttam, RahulScarano, AntonioGavrilović, AnaSchalkwyk, Antoinette VanGaweł-Bęben, KatarzynaSchalli, MichaelGawlik-Dziki, UrszulaScharf, BirgitGawronski, Stanislaw W.Scheer, HugoGazarini, Marcos LeoniSchejter, EduardoGazi, Anastasia D.Schill, Kristin M.Gebiola, MarcoSchirtzinger, Erin E.Genovese, FrancescoSchito, Anna MariaGenovese, Kenneth J.Schizas, DimitriosGenta, Fernando ArielSchlosser-Brandenburg, JosephineGentile, AntoninoSchmidt, JohannesGeorge, AndersonSchmidt, MarcinGeorgescu, CeciliaSchmidt, OlafGeorgieva, MargaritaSchmidt, VolkerGermán, Camacho-MorenoSchnütgen, FrankGharbi, JawharSchoen, Mary E.Ghenea, Alice ElenaSchröttner, PercyGherman, Calin MirceaSchubert, KristinGhiciuc, CristinaSchüler, LisaGhinea, CristinaSchumann, RhenaGiaccone, ValerioSchwaiger, KarinGiammanco, AnnaSchwarz, PatrickGianfredi, VincenzaSchwenk, JochenGiani, MicheleScopa, AntonioGiannenas, IliasScorpio, DianaGiantsis, Ioannis A.Scortichini, MarcoGiaouris, EfstathiosScott, IanGiardi, Maria Teresa EresaSeal, BruceGilles, AndréSebastian, Patrick StephanGillings, MichaelSecott, TimGilmore, DaveSedeik, Mahmoud E.Gilmour, Cynthia C.Sedlakova-Kadukova, JanaGilmour, MatthewSedlar, KarelGilpin, BrentSegala, FrancescoGimpel, MatthiasSegal-Kischinevzky, ClaudiaGinet, NicolasSekoai, PatrickGinevra, ChristopheSelim, Abdelfattah M.Gionchetta, GiuliaSemenkov, IvanGiovanneti, ManuelaSemenova, EkaterinaGiovannetti, ManuelaSenpuku, HidenobuGirault, GuillaumeSepúlveda, LeonardoGîrd, Cerasela ElenaSergiev, PetrGirdhar, KhyatiSerra, Cláudia R.Girigoswami, KoyeliSerra, GloriaGirolamini, LunaSerrano Andrés, María JesúsGisder, SebastianŠestanović, StefanijaGits-Muselli, MaudSethupathy, SivasamyGiudice, ElisabettaSeung-Hun, LeeGiurg, MirosławSeverns, Paul M.Gkelis, SpyrosSeydel, KarlGkizi, DanaiShadrin, AndreyGłąbska, DominikaShaheen, MohamedGlaeser, Stefanie P.Shahini, EndritGnanagobal, HajaroobaShang, QingsenGnat, SebastianShanmugam, Saravanan RamiahGniadek, AgnieszkaSharafutdinov, IrshadGniewosz, MałgorzataSharma, AnupamGobin, IvanaSharma, MinaxiGoda, Emad S.Sharma, PoonamGodfrey, Henry P.Sharshov, KirillGoel, RameshShastry, Rajesh P.Goethert, HeidiShaw, AndrewGoh, Kelvin G. K.Shcherbakov, DmitriyGołab˛, KrzysztofShcherbakova, ViktoriaGolan, KatarzynaShearwin, Keith E.Golba, SylwiaShehabeldine, AmrGolec, PiotrShehata, Awad A.Golubeva, TatianaShehata, MahmoudGolubkina, Nadezhda A.Shehata, Mohamed G.Gómez, FernandoShelat, Vishalkumar G.Gomez, José Gregório CabreraShelenkov, Andrey A.Gómez-Clavel, José F.Shelomi, MatanGómez-Junyent, JoanShen, ChingjuGómez-Mejia, AlejandroSher, FarooqGómez-Muñoz, María TeresaShi, YuGómez-Pinchetti, Juan LuisShi, ZhaoyongGómez-Villegas, PatriciaShiao, Young-JiGonçalves, ItamarShiekh, KhursheedGoncharenko, Anna V.Shih, Chun-KuangGong, JunShilev, StefanGong, SanqiangShimohata, TakaakiGongal, GyanendraShimosato, TakeshiGonzález García, VicenteShin, Seung GuGonzalez, GaelleShipitsyna, ElenaGonzález, Gloria M.Shirreff, GeorgeGonzalez, Juan M.Shivanna, VinayGonzález, MarioShoda, MakotoGonzález, Víctor M.Shrestha, NirajanGonzález-Barrio, DavidShridhar, Pragathi BelagolaGonzález-Cerón, LiliaShrivastava, SajalGonzález-Domínguez, IreneSi, FushengGonzález-López, ÓscarSiddiqui, ArifGonzález-Redondo, PedroSiddiqui, Mohammad TahirGopal, PramodSiddiqui, RuqaiyyahGoraj, WeronikaSidjabat, Hanna EvelinaGóral, DariuszSidorenko, SergeyGóral, TomaszSiebert, Hans-ChristianGorasia, DhanaSiegele, DeborahGormley, EamonnSieira, RodrigoGórniak, DorotaSierra-Campos, ErickGórska, Ewa BeataSiesto, GabriellaGosciniak, GrazynaSievers, SusanneGosiewski, TomaszŠigut, MartinGospodarek, JaninaSikder, Mohammad Nurul AzimGotovtsev, PavelŠikić-Pogačar, MajaGougoulis, Dimitris A.Sikora, MariuszGoujgoulova, GabrielaSikorski, ŁukaszGoytia, MairaSilva, Célia C. G.Grabarczyk, MałgorzataSilva, Maria DanielaGraczyk, RadomirSilva, Nathalia Cristina CironeGralak, MikołajSilva, RicardoGranados-Chinchilla, FabioSilva, Severino Jefferson Ribeiro DaGrande, RossellaSilva, Sónia CarinaGrandin, TempleSilva-Caso, Wilmer GianfrancoGrange, Philippe A.Silva-Costa, CatarinaGranica, SebastianSimbirtsev, AndreyGrass, GregorSimion, IsabelaGrassi, AusiliaSimões, ManuelGregorio, Simona DiSimões, Marta FilipaGrewal, JasneetŠimon, Martin C.Grieco, FrancescoSimonini, RobertoGrier, Jennifer T.Simpson, David J.Griesbeck, Axel G.Simpson, Thomas J.Grigoriev, AndreyŠimunović, KatarinaGrilli, EsterSinden, Robert E.Grillo, SaraSinderen, Douwe VanGrishin, Sergei Y.Singh, GurpreetGrobbel, MirjamSingh, ParbeenGroma, ValerijaSingh, RaveshGrosso, ClaraSingh, Sandeep KumarGrüber, GerhardSingh, TejGruber, James V.Sinha, DhritiGrygansyi, AndriiSinitsyna, Olga A.Grygorcewicz, BartłomiejSinzger, ChristianGrzesiak, JakubSipos, GyörgyGuembe, MaríaSiracusa, RosalbaGuérin, FrançoisSirekbasan, SerhatGuerra, ManuelaSkaro Bogojevic, SanjaGuerrant, RichardSkliros, DimitriosGuerreiro, Marco AlexandreSkorokhod, OleksiiGuerrini, AlessandroSkoufos, IoannisGuest, Johnathan D.Skowronek, MarcinGuevara González, RamónSkrlep, MartinGuha, MayukhSkrypnik, KatarzynaGuida, MarcoSkurnik, MikaelGuimarães, Maria AngelicaŚliwińska-Wilczewska, SylwiaGulla, KrishnaSłomka, ArturGulshani, AshkanSluder, Ann E.Guo, HaixinSmedile, FrancescoGuo, HongguangSmeti, EvangeliaGuo, KunSmigic, NadaGuo, XiaoleiSmith, KirstyGupta, PoojaSmith, Stephen A.Gupta, Radhey S.Smułek, WojciechGupta, Shishir KumarSnyder, ChristopherGupta, SushimSobel, JackGupta, YashSobhani, IradjGurgu, Leontina C.Sobral, Maria Do CarmoGursky, VitalySobrino-Plata, JuanGusev, OlegSoco, EleonoraGuterres, AlexandroSöderhäll, KennethGutiérrez Merma, AntonioSofia, MarinaGutierrez, Enriqueta GarciaSóki, JózsefGutiérrez, Juan CarlosSolano, FranciscoGutierrez, LiliaSolà-Oriol, DavidGuy, Rebecca A.Soldan, Samantha S.Guzik, UrszulaSolís García Del Pozo, JuliánHa, Nam-ChulSolopov, PavelHać-Szymańczuk, ElżbietaSolovchenko, AlexeiHaddad, NabilaSoltan, Mohamed A.Hadj Saadoun, JasmineSolyanikova, Inna P.Haese, Nicole N.Sombetzki, MartinaHaga, SatoshiSomerville, Greg A.Hagagy, NashwaSomeya, NobutakaHagemann, MartinSommerhalter, MonikaHagen, Ralf MatthiasSong, FuhangHagi, TatsuroSong, Myoung ChongHajdú, EditSong, YanyuHajfathalian, MaryamSoriano-Ursua, Marvin AntonioHalabowski, DariuszSorokan, AntoninaHale, JohnSosa Gomez, DanielHall-Mendelin, SonjaSosio, MargheritaHalvatsiotis, Panagiotis G.Soti, Pushpa G.Hammer, EdithSoto, Lorena PaolaHan, BingSousa Dos Santos, José CleitonHan, Dong-WookSousa, Sílvia A.Han, JiruŠovljanski, OljaHan, Sang-WookSowa, SylwiaHan, ZhenlinSpector, Stephen A.Hančević, KatarinaSperone, EmilioHanefeld, UlfSpiliopoulou, AnastasiaHao, JianjunSpiller, O. BradHao, LizhuangSpina, FedericaHao, ZikaiSpiridonov, SergeiHaque, Md HakimulSplichalova, AllaHardies, Stephen C.Spoto, SilviaHarja, MariaSridhar, KandiHaroutounian, Serkos A.Srikhanta, Yogitha N.Harris, Robert A.Srinuanpan, SirasitHarrison, Kelly S.Sroka-Oleksiak, AgnieszkaHaruna, TakenoriStaehelin, ChristianHasan, Nabeeh A.Štajner, TijanaHassan, Ahmed AbdelkhalekStalažs, ArtursHassan, Hosni M.Stamatikos, AlexisHassan, Saad El-DinStammes, MariekeHassani, Mohamed-AmineStancu, Mihaela MarilenaHawdon, John M.Stanek, AgataHawman, DavidStanimirovic, ZoranHayashi, ShuheiStaninska-Pięta, JustynaHayer, Shivdeep SinghStasiak-Różańska, LidiaHazard Taft, DianaStavropoulou, ElisavetHe, LeiStefan, Daniela SiminaHe, YinanStefania, TocciHeczko, PiotrStefanini, IreneHederstedt, LarsStefańska, IlonaHefferon, Kathleen L.Stefanski, TomaszHeider, JohannStein, DanielHeikkinen, Anna MariaSteinman, AmirHelmy, Yosra A.Steinmann, EikeHendiger, Edyta. B.Stendahl, OlleHeng, NicholasStepanova, AnnaHenry, Ajuna B.Stephens, DavidHerman-Bausier, PhilippeStephens, EdwardHernández Sánchez, HumbertoStępień, ŁukaszHernández-Martínez, RufinaSterkel, AlanaHerosimczyk, AgnieszkaSterniša, MetaHerrbach, EtienneStevens, David ColeHerrera, HectorStewart, HollyHerrera-Rincon, CeliaStewart, Jay M.Herrero Moreno, AntoniaStingl, KerstinHesham, Abd El-LatifStirbet, AlexandrinaHeumann, ChristianStocco, GabrieleHeyer, RobertStock, WillemHeyndrickx, MarcStoian, VladHietala, AriStopic, SreckoHiguchi, YujiroStracquadanio, StefanoHilário, SandraStraily, AnneHilbert, FriederikeStriker, Rob T.Hildebrand, JoannaStrle, FrancHiraishi, AkiraStromberg, ZacharyHiroyukuki, HaradaStrüder-Kypke, Michaela C.Ho, JamesStrychalski, JanuszHo, Thao T.B.Styczyński, JanHoffmann, BerndSu, XiaomeiHogan, Andrew M.Subedi, DineshHolb, ImreSubhadra, BinduHolbrook, MichaelSubramanian, ParthibanHole, Camaron R.Suchodolski, JakubHolík, LadislavSuda, ShoichiroHolland, Jason W.Suhrbier, AndreasHollister, Emily B.Sujkowska-Rybkowska, MarzenaHolmager, PernilleSulej, JustynaHoltmann, DirkSulo, PavolHomblé, FabriceSultana, HalimaHoneyborne, IsobellaSun, FengjieHong, Bo-youngSun, JunHong, Jiann-RueySun, XiaolunHong, Kwang WonSun, ZhichenHongbo, HuSung, WayHönig, VáclavSur, IoanaHopkins, Frances E.Suslo, RobertHoracek, MichaSuteu, DanielaHorhat, DeliaSutton, GrangerHorhat, Florin GeorgeSuvorov, Alexander NikolaevichHorianopoulos, Linda C.Suzui, NobuoHornung, BastianŠvedienė, JurgitaHorvath, ThomasSwami, DeepakHoshino, TamotsuSwart, ArnoHossain, Md. IqbalSwedberg, GöteHotchkiss, Arland TSweeney, EmmaHou, Chih-YaoŚwiątek, ŁukaszHou, Wen-ChiŚwiątkiewicz, MałgorzataHou, ZhishuaiSwietnicki, WieslawHouhamdi, LindaSwingley, WesleyHoyne, Gerard F.Syranidou, EvdokiaHrabar, JerkoSyridou, LiaHtoutou Sedlakova, MiroslavaSzablewski, TomaszHu, ChenlinSzafranek-Nakonieczna, AnnaHu, JieSzentiványi, TamaraHu, WeiSzili-Kovács, TiborHu, XiaoSzponar, BogumiłaHuang, BoSzulc-Dąbrowska, LidiaHuang, Chien-JuiSzweda, PiotrHuang, I-HsiuSzydlowska, AleksandraHuang, JiachenSzymańska-Czerwińska, MonikaHuang, MingtaoTabacchioni, SilviaHuang, ZhuangrongTabain, IrenaHugerth, Luisa WarchavchikTabish, Tanveer AhmadHugouvieux-Cotte-Pattat, NicoleTaddei, SimoneHuh, Sung UnTagliavia, MarcelloHukkanen, VeijoTaha, El-Kazafy AbdouHume, Michael E.Taha, Muhamed KheirHung, Shao-WenTaibi, AmelHuntosova, VeronikaTakamatsu, DaisukeHuseien, Ghasan FahimTakeda, ShigekiHussain, MubasherTakenaka, YasuhiroHussein, HalaTakeno, SachioHussein, MaythamTaki, RegraguiHutchings, MichaelTakors, RalfHutorowicz, AndrzejTalà, AdelfiaIannelli, FrancescoTalebi Bazmin Abadi, AminIanni, AndreaTalmon, MariaIbaba, Jacques DavyTalon, RegineIbrahim, AbdallahTalukdar, Prabhat K.Ibrahim, SalamTamang, PrabinIbrahimi, ManarTamba, MarcoIerodiakonou, DespoTamhankar, ManasiIgarashi, YasuhiroTan, Bee KangIgnatov, AlexanderTan, ZhongfangIkoyi, IsraelTanaka, MizukiIlic, NebojsaTanase, Daniela MariaIliescu, Florina S.Táncsics, AndrásIlyas, SadiaTandukar, SarmilaIlyasov, RustemTang, JialingImhoff, JohannesTang, Ri-YuanImre, KálmánTang, Tzu-ChiehImre, MirelaTang, XiangmingInamdar, ShivangiTangadanchu, Vijai Kumar ReddyInês, AntónioTania, Martín-PérezIngham, Colin J.Tao, XuanyuIngwell, Laura L.Tao, YongInokuchi, RyotaTaoka, YousukeInoue, ChihiroTarcz, SebastianInoue, DaisukeTardy, FlorenceIoannou, PetrosTarka, PatrykIorga, BogdanTarrah, ArminIorizzo, MassimoTassone, SoniaIqbal, MudassirTate, HeatherIsaacs, ArielTaus, NaomiIsaeva, Marina P.Tausch, Simon H.Isakova-Sivak, IrinaTawfick, Mahmoud MohamedIshii, DaisukeTaylor, MatthewIshikawa, TakaoTaylor, Thomas MatthewIshioka, TaiseiTeixeira, PilarIslam, M. AmirulTelatin, AndreaIslam, RabiulTelegdi, JuditIsokpehi, RaphaelTeleky, Bernadette EmokeItabashi, KazuoTéllez-Valencia, AlfredoIto, KazuoTemesvari, LeslyIturriaga, TeresaTeneva, IvankaIvančić Šantek, MirelaTeng, Jade Lee LeeIvancic-Bace, IvanaTenhagen, Bernd-AloisIvankovic, TomislavTeo, AndrewIvanov, IliyanTeper, DoronIvanov, MarijaTerekhov, Stanislav S.Ivanova, Anastasia A.Terzić-Vidojević, AmarelaIvanova, IlianaTetaud, EmmanuelIvshina, IrenaThanki, Anisha MahendraIwagami, MoritoshiThaochan, NaritIwasaki, YukiThapa, JeewanIzadi, PanizTharappel, AnilIzdebska, Joanna N.Thébault, AnneIzzi, MargheritaTheologidis, IoannisJacquet, StephanThiel, VeraJafarinejad, ShahryarThipe, Velaphi C.Jailani, Abdul KaderThissera, BathiniJain, SwatiThitiananpakorn, KanateJakovac-Strajn, BredaThomas, Roger E.Jakubczyk, KarolinaThompson, PeterJakubska-Busse, AnnaTian, LingJames, Claire D.Tibpromma, SaowaluckJanalikova, MagdaTihăuan, Bianca MariaJang, JeonghwanTikhe, Chinmay V.Jang, Jong-HwaTimenetsky, JorgeJang, Tyng YuanTimmons, Jennifer RumseyJankiewicz, UrszulaTinebra, IleniaJanusz, GrzegorzTirado-González, Deli NazmínJasbi, PanizTiscar, Pietro G.Jass, JanaTischler, DirkJaswal, RajneeshTisi, RenataJaved, MohsinTit, Delia MirelaJaworski, RadoslawTjernberg, IvarJeddi, FakhriTkachenko, OksanaJenkins, AndrewTkaczyk, MiłoszJensen, Per MoestrupTodorov, SvetoslavJeong, HanseobTodorova, DessislavaJerônimo, Gabriela TomasToffanin, AnnitaJessen-Trefzer, ClaudiaTomas, MatejJesudoss Chelladurai, JebaTomaszewska, EwaJia, FangxuTomczuk, KrzysztofJia, WeihuaTomczyk, ŁukaszJia, XiaojiongTomić, AnaJiang, CancanTomoda, HiroshiJiang, GuangdeTong, Yen WahJiang, HongToro, NormanJiang, HongchenTorzewska, AgnieszkaJiang, ShengjingTosi, SolveigJiang, XiaobingToth, ZoltanJiang, YuemingTouliabah, HusseinJiao, HanweiTouloupakis, EleftheriosJiao, JunyiToya, YoshihiroJiménez Ballesta, RaimundoTrabaud, Mary‐AnneJimenez Bremont, Juan FranciscoTraina, GiovannaJiménez Gómez, Pedro A.Tralhão, AntónioJiménez-Ruíz, YolandaTrček, JanjaJin, ChengTrdan, StanislavJin, LongTrevisan, GiustoJoachim, AnjaTrifonova, Tatiana A.Johansen, MattTrifuoggi, MarcoJohansson, BjörnTrombetta, CarloJohnson, KristenTrotta, VincenzoJohnson, Michael D. L.Trovão, JoãoJohnson, NicholasTruncaitė, LidijaJokovic, NatasaTrusek, AnnaJones, BrianTsai, Ying-MingJones, MarjorieTsamesidis, IoannisJordao, LuisaTseng, Jen-ChihJoshi, Lovleen TinaTsivileva, OlgaJouda, Jean-BoscoTsouknidas, AlexanderJovanović, MarinaTsui, To-HungJuliano, Claudia Clelia AssuntaTsuji, MasaharuJung, ChuleuiTsukatani, YusukeJung, SokheeTu, Tzu-HsuanJung, TaesungTufarelli, VincenzoJunior, Mario LiraTufariello, MiriamJuráček, MiroslavTullio, VivianJurado, Macarena M.Tulsian, Nikhil K.Jurasek, MichalTundo, SilvioJuste, CatherineTung, Yen ChenKačániová, MiroslavaTunsjø, HegeKačergius, AudriusTuovinen, OlliKaczmarek, AgataTurkewitz, Aaron P.Kaden, ReneTurowski, GittaKagambèga, AssètaTurton, Jane F.Kahler, CharleneTurudić, DanielKahlert, GuentherTutukina, Maria N.Kaiser, RolfTyubaeva, PolinaKajak-Siemaszko, KatarzynaTyurin, AntonKajsík, MichalTzeng, Tzuen-RongKaldhone, PravinTziros, George T.Kalinichenko, AntoninaTzora, AthinaKalisz, StanisławUebanso, TakashiKalita, MichałUeda, AkihiroKalmár, ZsuzsaUhlich, GaylenKaltschmidt, BarbaraUhrynowski, WitoldKalyuzhnaya, MarinaUjor, VictorKamali, MohammadrezaUldum, SorenKambourova, MargaritaUllah, FazalKaminski, Tomasz W.Ullah, RiazKamli, Majid RasoolUmemura, MasayukiKamysz, WojciechUndersander, DanKamzolova, SvetlanaUpdegrove, Taylor B.Kanakis, IoannisUrban-Chmiel, RenataKanelis, DimitriosUrbańczyk-Lipkowska, ZofiaKanetis, LoukasUrbański, ArkadiuszKang, Ji YoungUrbina, HectorKang, JinheUrlić, BranimirKang, MingsongUspensky, IgorKang, SanghoonUssetti Gil, PiedadKanipe, CarlyUsui, TatsufumiKano, RuiUte, Risse-BuhlKansau, ImadUversky, VladimirKant Bhatia, ShashiUyeno, YutakaKantere, Maria C.Uzuhashi, ShihomiKao, Cheng-YenVadkertiová, RenátaKapetanović, DamirVale, Filipa F.Kaplan, ShaunaValenzuela, Rodrigo WladimirKapusta, PiotrValeriani, FedericaKarabasil, NeđeljkoValkov, VladimirKaraduta, OlegVan Den Berg, Marco AlexanderKaraffova, VieraVan Der Vossen, Jos M. B. M.Karakonstantis, StamatisVan Rhijn, NormanKaranis, PanagiotisVan Straalen, Nico M.Karaś, MagdalenaVankadari, NaveenKarki, BishnuVannucchi, Maria-GiulianaKarliński, LeszekVanwonterghem, InkaKarlsson, RogerVargas-García, María Del CarmenKarpiński, Tomasz M.Varriale, SimonaKarthick Raja Namasivayam, SelvarajVarzakas, TheodorosKarvouniaris, MariosVasić, Tanja P.Kasai, DaisukeVasina, Daria V.Kashtanova, DariaVasquez, IgnacioKasote, DeepakVasquez-Rifo, AlejandroKatapodis, PetrosVázquez Belda, Beatriz I.Kathayat, DipakVázquez-Marrufo, GerardoKatinakis, PanagiotisVazquez-Munoz, RobertoKato, HirotomoVeerana, MayuraKatoh, HiroshiVega-Vera, Francisco J.Katsafadou, AngelikiVeiga Junior, Valdir F.Katsaras, GeorgiosVeiga, Maria IsabelKaushik, PrashantVeiga, SilvioKavita, KumariVelazquez-Salinas, LauroKawada-Matsuo, MikiVelikova, TsvetelinaKawaguchi, AkiraVeloo, AlidaKawaguchiya, MitsuyoVenken, Koen J. T.Kawamoto, EijiVenturini, MarianoKawamura, YoshikiVenus, JoachimKaye, Stephen B.Verganud, GillesKazachenko, AlexandrVergkizi-Nikolakaki, SouzanKazimírová, MariaVerhoeven, JoostKe, ShanlinVerma, VinodKędziora, AnnaVerri, TizianoKeiffer, TimothyVicente, Giner GalvánKeller, SandroVickery, KarenKelly, BenderVidal, PiaKemp, StevenVideau, PatrickKenney, ShannonVidican, RoxanaKenny, JohnVieira, YasminKenzaka, TsuneakiVigliotta, GiovanniKeros, TomislavVijayakumar, SekarKerr, Edward D.Vílchez, Juan IgnacioKeswani, ChetanVilibić-Čavlek, TatjanaKhalifa, AshrafVilla, LucaKhalloufi, Fatima ElVillamor, Miguel Ángel CastroKhan, Aman UllahVillanueva, MarcoKhan, AsisVillarruel-López, AngélicaKhan, FazlurrahmanVillaverde, JaimeKhan, Munawwar AliVillella, Valeria RachelaKhan, Raja Asad AliVimercatti, LaraKhmelenina, Valentina N.Viñas, MiguelKhona, DollyVinti, GiovanniKhor, Wei ChingVintila, TeodorKibirige, Catherine N.Virella, DanielKiczorowska, BożenaVirgínio Júnior, Gercino FerreiraKidd, Stephen P.Vishnyakov, Innokentii E.Kidron, GioraVissink, ArjanKieffer, NicolasVitale, MariaKieliszek, MarekVito, PasqualeKilk, KalleVitor, RamosKilstrup, MogensVitti, AntonellaKim, ChoonViuda-Martos, ManuelKim, HeejungViver, TomeuKim, Hei-sungVlaicu, AlexandruKim, HoonVlamis-Gardikas, AlexiosKim, Hwa-JungVlasov, DmitryKim, Hye KwonVlok, MarliKim, Hye OneVogt, SebastianKim, Hyun-oukVoidarou, Chrissoula (Chrysa)Kim, In JungVon Ah, UeliKim, Jae-SeokVon Fricken, MichaelKim, Jin IlVon Knethen, AndreasKim, JungeunVoronina, Olga L.Kim, KyuseokVrchotová, BlankaKim, SeilVrioni, GeorgiaKim, Seung IlVulturar, RomanaKim, Soo-KiVyzantiadis, Timoleon-AchilleasKim, Young-KeeWadas, WandaKimbrel, JeffreyWadhwani, ParveshKing, JacquelineWadsö, LarsKinoshita, YutaWagner, GabrielKinthada, LakshmanakumarWahab, TaraKirchberger, PaulWahed, Ahmed Abd ElKirchgatter, KarinWakiguchi, HiroyukiKirillov, Alexander A.Wakuliński, WojciechKitaeva, AnnaWalczak, MarekKittl, SonjaWalker, JenniferKlčová, LenkaWall, Richard J.Klutsch, CornelyaWalochnik, JuliaKlutsch, JenniferWalsh, Laurence JKlymiuk, IngeborgWan, YanminKnauf, SaschaWanchai, VisanuKnuepfer, EllenWang, ChengliangKo, Kwan SooWang, HsiuyingKo, Seok-jaeWang, JiezhongKobae, YoshihiroWang, JinquanKobayashi, KazuoWang, KaiKobayashi, NobumichiWang, LeiKobierzycki, ChristopherWang, LuguangKoblizek, MichalWang, MinKoch, Hans GeorgWang, Ming-ChengKocot, AleksandraWang, PenghaoKocot, Aleksandra MariaWang, QiKocsis, BelaWang, QimingKodym, PetrWang, QinzheKoeffel, ReneWang, Robert Yung LiangKoga, YasuhiroWang, ShianKoh, Sung-CheolWang, ShuangKoide, Roger T.Wang, TongKojic, MilanWang, WeiKoksharova, Olga A.Wang, WenjiaKokubu, EitoyoWang, XinKolář, MilanWang, XuKollia, PanagoulaWang, XudongKollman, Justin MWang, YangKolmeder, CarolinWang, YunshanKołodyńska, DorotaWang, ZhixueKołodziej, BogusławWareth, GamalKołodziej, PrzemysławWarner, DouglasKolomeichuk, SergeyWatanabe, MaiKolpa, MalgorzataWawrocki, SebastianKomaki, HisayukiWdowiak-Wróbel, SylwiaKonishi, MasaakiWeber, JanKonjar, ŠpelaWęgrzyn, GrzegorzKonstantakis, ChristosWei, DongqingKonstantinidis, Konstantinos T.Wei, XiaoyuanKonteles, SpyrosWei, YuqiuKoo, Byung-SooWeichhart, ThomasKooistra, Wiebe H.C.F.Weidmann, ManfredKopra, KariWeigmann, SimonKorac, MilosWeiland-Bräuer, NancyKorać, PetraWeis, Allison M.Koraimann, GüntherWerszko, JoannaKormas, Konstantinos Ar.Weyrich, LauraKorona-Glowniak, IzabelaWheeler, TerryKorshun, Vladimir A.Whisnant, Adam W.Korshunova, TatianaWhite, Aaron P.Korzekwa, KamilaWhitledge, Terry E.Korzhenkov, A. A.Wick, MacdonaldKosakyan, AnushWidelski, JarosławKoshizuka, TetsuoWidemann, EmilieKostecka-Gugała, AnnaWiench, RafałKostrzewa, MarkusWiewióra, BarbaraKostyanev, TomislavWilhelm, Roland C.Kot, Anna MariaWilk, MateuszKot, KarolinaWilletts, AndrewKotlowski, RomanWilliams, Rohan B. H.Kotta-Loizou, IolyWillows, Robert DrantKottaridi, ChristineWilusz, JeffKouhsari, EbrahimWiniszewska, GrażynaKouokam, Joseph CalvinWinkler, JanKoupaie, EhssanWinklhofer, Michael WinklhoferKourkoutas, IoannisWitkowski, LucjanKoutoulis, KonstantinosWohlleber, DirkKouzai, YusukeWojnicz, DorotaKováč, JánWojtyczka, RobertKovač, MarijaWolna-Maruwka, AgnieszkaKowalczyk, PawełWolny-Koładka, KatarzynaKowalski, TadeuszWon, Eun JeongKozak, AnnaWong, Antonio Cheuk PuiKozera, WojciechWong-Villarreal, ArnoldoKozłowska, JoannaWouw, Marcel Van DeKrabel, DorisWoźniak-Karczewska, MartaKranjec, ChristianWoźniakowski, GrzegorzKranz, StefanWrotek, AugustKrasnopolskaya, LarissaWu, DandanKrasowska, AnnaWu, NanKrause-Sorio, BeatrixWu, PengKrauze, MagdalenaWu, ZeniKrawczyk, BeataWu, ZhenbingKrawczyk, KrzysztofWu, ZhengyunKrešić, NinaWultańska, DorotaKrisch, JuditWydro, UrszulaKrishnamurthi, Venkata RaoWysok, BeataKrivopalov, AntonWyszkowska, JadwigaKrivoruchko, AnastasiiaXia, ZhichaoKroiča, JutaXiang, XianlingKrol, BarbaraXiao, Jin-zhongKróliczewska, BożenaXiao, SunKrug, LaurieXiao, WeikunKruger, AndreasXiao, YongliKrüger, NadineXie, Hua-TaoKrukowski, HenrykXie, JianghuiKruszewska, JoannaXie, YucenKrzysztof, AnuszXie, YuyuanKrzywonos, MałgorzataXie, ZhengjunKsiazczyk, MartaXimenes, EduardoKu, Yee-ShanXimenez, CeciliaKubera, ŁukaszXing, JingKubiak, KatarzynaXu, JianKubiak, PiotrXu, JingjingKucharski, JanXu, QingbiaoKučić Grgić, DajanaXu, YuanweiKučinskaite-Kodze, IndreXue, HonghaiKuczius, ThorstenXue, LinguiKudinova, Alina G.Xun, LuyingKudryashov, Dmitri S.Yabaji, ShivrajKuever, JanYadav, VirendraKuhn, RamonaYahya, EsamKulakovskaya, Tatiana V.Yamaguchi, MasahikoKumar Singh, RajeshYamamoto, TomofumiKumar, AjayYang, ChuanbinKumar, AnandYang, Chul-SuKumar, DeepakYang, FengxiaKumar, Manish VijayaYang, GuangKumar, MuruganYang, HuaiyuKumar, RavinderYang, MingKumar, SantoshYang, QingKumaraswami, KondaYang, Tsung-YingKunova, AndreaYang, Yu-liangKuo, Chia-HungYangcheng, LuKurakula, MalleshYao, QingxinKurita, JunYaparla, AmulyaKušar, DarjaYaqoob, Asim AliKuwahara, TomomiYaroslavtseva, OlgaKuźniar, AgnieszkaYassin, AymenKwiatkowski, Cezary A.Yassin, MohamedKwon, Oh-DeogYavitt, JosephKwun, Hyun JinYellapu, Nanda KumarKyaw Thu, AungYeom, JinkiKyndt, JohnYeremeev, VladimirKyriakopoulos, Anthony M.Yerushalmi, LalehKyriazopoulou, EvdoxiaYing, BeiwenKyritsi, MariaYokoyama, YokoLa Bella, GianfrancoYon, Dong KeonLa Russa, FrancescoYoon, SunghyunLaabei, MaisemYoshida, NaotoLabrou, NikolaosYoshihiro, SuzukiLacey, HeatherYou, XiaomengLaczi, KrisztiánYounis, Nancy S.Lad, ApurvaYoussef, MahmoudLade, Harshad S.Youssef, Noha H.Ladomenou, FaniYoussef, Sahar A.Lager, MalinYu, Fu-QiangLagogiannis, IoannisYu, JunpingŁagowski, DominikYu, Tsz TinLaGrow, Austin L.Yu, Wen-LiangLai, Kuei-HungYu, YaochunLai, OlimpiaYuan, LinxiLajqi, TrimYuan, PeiguoŁakomy, PiotrYuan, Shuo-FuLal, Charitharth VivekYuan, ZihaoLamas, AlexandreYuanyuan, WangLambert, Jean-PhilippeYurchenko, EkaterinaLambert, JoYurimoto, HiroyaLammers, Peter J.Yutaka, YoshiiLaMontagne, MichaelZaapala, BarbaraLampridis, SavvasZabłocka-Godlewska, EwaLane, Hsien-YuanZaborowska, MagdalenaLang, ChristineZacharias, NicoleLaplace, Jean-MarieZachwieja, AndrzejLaranjo, MartaZadravec, ManuelaLarkum, AnthonyZafar, HassanLasik-Kurdyś, MałgorzataZago, MiriamLaskowska, EwaZaheer, RahatLasonder, EdwinZaidi, Syed FaisalLasseur, ChristopheZaini, PauloLastauskienė, EglėZakalyukina, Yuliya V.Lastochkina, OksanaZakrzewski, ArkadiuszLatini, AriannaZammuto, VincenzoLatosińska, JolantaZamorska, JustynaLaura, Martinez-AlvarezZampolli, JessicaLauretani, FulvioZan, FeixiangLawal, BashirZanchi, CarolineLazar, Cassandre S.Zanfardino, AnnaLazar, CatalinZaragoza, WilliamLazarević, JelenaZarubaev, Vladimir V.Lazarevic-Pasti, TamaraZavyalova, ElenaLazarova, StelaZawierucha, KrzysztofLazic, VesnaZdarta, AgataLe Bars, PierreZdolec, NevijoLe Doaré, KirstyZdrazilova‐Dubska, LenkaLe, ShuaiZębek, ElżbietaLeardini, DavideZegeye, Ephrem DebebeLebre, PedroZeier, MartinLechuga, SusanaZekker, IvarLeclerque, AndreasZelena, LiubovLedford, Julie G.Zeleny, ReinhardLee, ChangguZell, RolandLee, Chang-SeokZeman, PetrLee, Che-HsinZendo, TakeshiLee, DongminZeng, MelodyLee, Hae KyungZeng, ZhengLee, JaslynZerva, AnastasiaLee, Je ChulZervas, AthanasiosLee, Jeong YoonZhai, YinLee, Sang-HanZhan, Xiao-YongLee, SangmiZhang Newby, Bi-minLee, SeungjunZhang, BaiyuLee, Sung-JaeZhang, DaweiLeer-Buter, Coretta VanZhang, DiLefort, FrançoisZhang, ErshuaiLegaz, IsabelZhang, FanLegler, PatriciaZhang, FenghuaLegorreta, MarthaZhang, GongLegros, VincentZhang, GongyiLehenbauer, Terry W.Zhang, JileiLehnherr, HansjörgZhang, KaiLei, ChaoZhang, LinglinLei, WangZhang, LongxianLeira, ManelZhang, PeilinLeitão, Jorge H.Zhang, PeipeiLejman, AgnieszkaZhang, RuiyongLeles, DanielaZhang, TingLemrani, MeryemZhang, WenjunLenz, VolkerZhang, WuchangLeong, Yoong KitZhang, XuehongLeon-Juarez, MoisesZhang, XueqinLeonova, Galina N.Zhang, YanjiangLeontopoulos, StefanosZhang, YifeiLepetit, MarcZhang, YinglaoLephart, Edwin D.Zhang, YongLepiarczyk, EwaZhang, Yu-QinLesterlin, ChristianZhang, ZhimingLestinova, TerezaZhao, DongminLettini, TeresaZhao, FangzhuLeuko, StefanZhao, SiyanLevican, ArturoZhao, XuboLevin, DavidZhao, YangguoLevizou, EfiZhao, YuejuLewandowicz, JacekZhelezova, Alena D.Lewis-Ximenez, Lia L.Zheng, ZhengLi, CharlesZhong, JinLi, Chien-FengZhong, Nai-QinLi, DongfangZhong, ZengtaoLi, DongmeiZhong, ZhipingLi, DunhaiZhou, ManLi, FuliZhou, TieliLi, HuashouZhou, TingLi, JinlinZhou, XiaohuiLi, Ju-PiZhu, LifengLi, LinZhukov, Vladimir A.Li, Po-HsienZiarno, MałgorzataLi, QiangqiangZidovec-Lepej, SnjezanaLi, QihanZielińska, DorotaLi, ShaojunŻielonka, ŁukaszLi, ShaotingZiembik, ZbigniewLi, TinggangZimenkov, Danila V.Li, YongcaiZingl, FranzLiang, CuiyueZissel, GernotLianou, AlexandraZlatkov Kolev, NikolaLiao, Chien-SenZłoch, MichałLiao, Wei-BiaoZmora, PawelLiao, Yu-ChiehZnamirowska, AgataLiaw, Shwu-JenZommiti, MohamedLiberato, Claudio DeZou, HuibinLiburdi, KatiaZubairova, UlyanaLicciardello, GraziaZubitur, Manoli M.Licker, Monica SorinaŽučko, JuricaLiduino, Vitor SilvaZuorro, AntonioLieber-Tenorio, ElisabethZygner, WojciechLiévin-Le Moal, Vanessa



